# Serum levels of SIRT3 in elderly patients undergoing general anesthesia and its relationship with postoperative delirium

**DOI:** 10.1515/med-2025-1359

**Published:** 2026-07-07

**Authors:** Linfeng Li, Huai Zhang, Rong Qian, HuiJuan Wang, Min Zhang, Lu Tang, Min Liao

**Affiliations:** Anesthesiology and Operating Room Center, The Third People’s Hospital of Chengdu, Affiliated Hospital of Southwest Jiaotong University, Chengdu, Sichuan, P.R. China

**Keywords:** postoperative delirium, general anesthesia, SIRT3, risk factors

## Abstract

**Objectives:**

To investigate whether serum SIRT3 levels can predict postoperative delirium (POD) in elderly patients undergoing general anesthesia.

**Methods:**

A prospective observational study was conducted on 118 elderly patients who underwent general anesthesia from January 2021 to December 2023. POD was assessed using the Confusion Assessment Method (CAM) within 72 h post-surgery. Serum SIRT3, IL-6, IL-1β, IL-17, and CRP levels were measured by ELISA. Clinical data including surgical time, anesthesia time, and blood loss were recorded. Statistical analysis was performed using SPSS 25.0 (p<0.05 considered significant).

**Results:**

Compared to non-POD patients, those with POD had longer surgery and anesthesia durations, lower hemoglobin and albumin levels, significantly lower serum SIRT3, and higher IL-6, IL-1β, and IL-17 levels. Serum SIRT3 was inversely correlated with IL-1β and IL-17. ROC analysis suggested SIRT3 as a potential predictive biomarker for POD. Logistic regression identified low SIRT3, low albumin, and elevated IL-1β and IL-17 as risk factors.

**Conclusions:**

Serum SIRT3 is significantly reduced in elderly POD patients and may serve as a predictive biomarker and potential therapeutic target for POD.

## Introduction

Postoperative delirium (POD) is a common and serious perioperative complication, often seen in elderly patients. POD is characterized by a significant decrease in attention, reduced ability to recognize the external environment, and pathological emotional disturbances [1], 2]. The incidence of POD in patients over 60–70 years of age is approximately 10–20 % [1]. Various hypotheses have been proposed to elucidate the mechanisms of POD, including oxidative stress, autophagy dysfunction, synaptic impairment, and lack of neuronal support [3], 4]. Among these, the neuroinflammation hypothesis is a more convincing one [5]. Previous studies found that abnormal serum cytokines in patients with POD [6], 7]. However, more serum biomarkers need to be explored to predict the occurrence of POD.

The sirtuin (SIRT) family plays various roles in different physiological and pathological events, including neurodegenerative diseases, age-related diseases, obesity, heart disease, inflammation, and cancer [8]. Among them, SIRT3 is primarily localized in mitochondria and regulates the deacetylation of non-histone substrates, particularly those involved in mitochondrial function and metabolism [9]. In recent years, SIRT3 has been found to play a role in regulating inflammation as a post-translational regulator, through the deacetylation of mitochondrial target proteins, in various inflammatory-related diseases [10]. Moreover, increasing evidence suggests that SIRT3 plays a significant physiological and pathological role in age-related diseases, particularly through its regulation of mitochondrial homeostasis and oxidative stress [11], 12]. Recent studies have shown that SIRT3 protects against postoperative cognitive dysfunction by activating AMPK/mTOR pathway-mediated autophagy, suggesting that activating SIRT3 may provide a new therapeutic approach to prevent postoperative delirium, a postoperative sequela [13].

Therefore, we aim to measure the serum levels of SIRT3 in elderly patients undergoing general anesthesia surgery and collect other clinical outcomes to investigate the predictive role of serum SIRT3 in the occurrence of POD in elderly patients.

## Methods

### Participants

This prospective observational study included 118 elderly patients who underwent general anesthesia surgery from January 2021 to December 2023. All patients were over 65 years old and underwent general anesthesia laparoscopic abdominal surgeries under the same medical team in our hospital. The exclusion criteriawere: 1) American Society of Anesthesiologists (ASA) grade III or higher; 2) patients with severe infection, brain disorders, or a history of mental or neurological diseases; 3) patients who were unable to complete the Mini-Mental State Examination (MMSE) assessment due to language, visual, or auditory impairments; 4) patients with preoperative MMSE scores ≤23; 5) patients who experienced severe complications intraoperatively or postoperatively; 6) patients who received anti-inflammatory or immunosuppressive treatments preoperatively.

To assess the perioperative cognitive status, all patients completed the Mini-Mental State Examination (MMSE) one day before surgery, and only patients with MMSE scores >23 were enrolled. In terms of delirium prevention strategies, all patients received standardized perioperative care, including adequate pain control, maintenance of normal sleep–wake cycles, orientation communication by nursing staff, early postoperative mobilization, and environmental optimization (minimizing nighttime noise and ensuring proper lighting). No prophylactic pharmacologic agents such as antipsychotics or melatonin were administered.

All patients underwent laparoscopic abdominal surgeries (including laparoscopic cholecystectomy, hepatic cyst fenestration, and partial hepatectomy) performed by the same surgical team.

### Anesthetic management

All patients received total intravenous anesthesia using propofol and remifentanil. Anesthesia was induced with propofol (1.5–2.5 mg/kg) and remifentanil (0.1–0.2 μg/kg/min), and maintained with continuous infusions of propofol (3–5 mg/kg/h) and remifentanil (0.1–0.2 μg/kg/min). Muscle relaxation was achieved with cisatracurium. No inhalational anesthetics were used. The depth of anesthesia was continuously monitored using the bispectral index (BIS, Covidien, USA) and maintained between 40 and 60 throughout surgery to ensure comparable intraoperative hypnotic levels.

### Diagnosis of POD

All elderly patients were evaluated for POD twice daily (at approximately 9:00 a.m. and 6:00 p.m.) within the first 72 h after surgery using the Confusion Assessment Method (CAM) [14] by a single anesthesiologist who was blinded to the patients’ serum biomarker data. The assessor underwent formal CAM training and certification prior to study initiation to ensure the accuracy and consistency of POD diagnosis. To minimize inter-rater variability, only one trained anesthesiologist performed all CAM assessments throughout the study. CAM includes four clinical features: (a) acute onset of cognitive changes and fluctuating course; (b) inattention; (c) disorganized thinking; and (d) altered level of consciousness. A diagnosis of POD was made when both features (a) and (b), and either (c) or (d), were present. Each assessment lasted approximately 10 min in a quiet ward environment, with verbal interaction and brief cognitive tasks. POD was defined as ≥1 CAM-positive assessment within this 72 h window.

### Enzyme-linked immunosorbent assay

Venous blood samples (5 mL) were collected at 24 h after surgery in the morning (approximately 7:00–8:00 a.m.) after an overnight fast. The levels of serum SIRT3, IL-6, IL-1β, IL-17, and CRP were measured using enzyme-linked immunosorbent assay (ELISA). Blood samples were centrifuged at 2000 *g* for 15 min, and the concentrations of these markers were determined with commercial assay kits (SIRT3-MBS163385, IL-6-MBS2021124, IL-1β-MBS2021180, IL-17-MBS2019491, and CRP-MBS2021863, MyBioSource, California, USA).

### Observational measures

Prior to surgery, we collected clinical and demographic data of all subjects, including sex, age body mass index (BMI), diastolic blood pressure (DBP), systolic blood pressure (SBP), ASA classification, and nutritional indicators, hemoglobin (Hb), albumin (ALB). Additionally, we recorded the surgical time, anesthesia time, and intraoperative blood loss for all patients.

The anesthesia duration was defined as the period from induction to extubation, while the surgery duration was defined as the time from skin incision to wound closure. The intraoperative blood loss was recorded by the anesthesiologist based on suction volume and gauze weight.

### Statistical analysis

Data analysis was performed using SPSS 26.0 (IBM, Armonk, NY, USA). The normal distribution of data was confirmed by Kolmogorov-Smirnov analysis. The Mann-Whitney U test or Student’s t-test was used to compare two groups. The chi-square test assessed proportions. Pearson’s correlation analysis evaluated the relationship between serum inflammatory markers in patients. ROC curve analysis was used to determine SIRT3’s ability to predict POD in elderly patients under general anesthesia. Logistic regression analysis identified risk factors for POD in these patients. A p-value of less than 0.05 was considered statistically significant.

This study has obtained approval of the Ethics Committee of The Third People’s Hospital of Chengdu and the ethical approval from the hospital’s ethics committee (No. CDTPH202107).

## Results

### Baseline data of all subjects

We included 118 elderly patients undergoing abdominal general anesthesia surgery. Within the first 72 h postoperatively, 33 of 118 patients (28.0 %) had at least one CAM-positive assessment (i.e., POD). Among patients with POD, 19/33 (57.6 %) were female compared with 45/85 (52.9 %) in the non-POD group; the sex distribution did not differ significantly (p=0.509). As shown in Table 1, the POD group had significantly increased anesthesia time and surgery time (Table 1, p<0.05). Additionally, patients in the POD group had significantly lower levels of Hb and ALB.

**Table 1:** Clinical characteristics of all participants.

Variable	POD patients, n=33	Non-POD patients, n=85	p-Value
Age, year	77 (65–89)	75 (65–85)	0.440
Sex, female (%)	19 (57.58)	45 (52.94)	0.509
BMI	22.61 (19.31–25.51)	23.01 (19.19–25.51)	0.606
SBP	129.89 ± 12.83	131.37 ± 14.19	0.604
DBP	83.36 ± 9.14	83.16 ± 8.76	0.913
Intraoperative blood loss, mL	34.59 ± 7.41	34.35 ± 7.35	0.681
ASA classification	2 (1–3)	2 (1–3)	0.446
Anesthesia time, min	176.36 ± 23.13	163.09 ± 20.11	0.003
Surgical time, min	152.53 ± 18.89	143.68 ± 18.22	0.021
Hb, g/L	100.18 ± 10.20	105.61 ± 11.51	0.019
ALB, g/L	32.81 ± 2.92	36.99 ± 3.25	<0.001

### Differential levels of serum markers in POD patients

**Figure 1:**
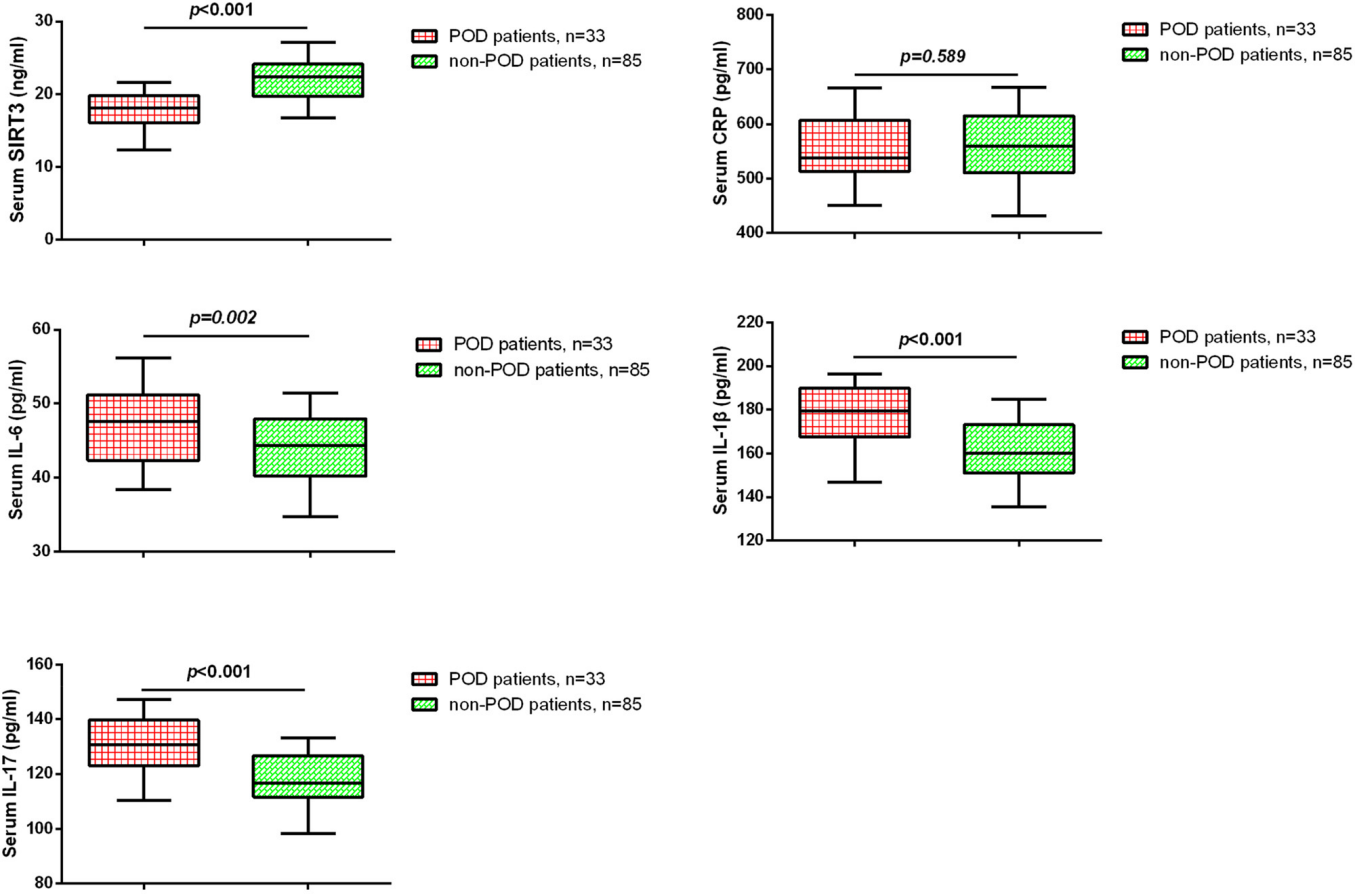
Serum SIRT3 and cytokines levels in POD patients.

Next, we measured the serum levels of SIRT3, iIL-6, IL-1β, IL-17, and CRP in elderly patients after general anesthesia surgery. As shown in Figure 1, POD patients had significantly lower levels of serum SIRT3. Moreover, POD patients showed significantly enhanced levels of serum IL-6, IL-17, and IL-1β compared to non-POD patients. There were no differences observed in the serum CRP levels between the two groups. Pearson’s correlation analysis revealed a negative relationship between serum SIRT3 levels and the levels of IL-1β and IL-17 (Table 2).

**Table 2:** Pearson’s correlation analysis among cytokines.

	SIRT3	CRP	IL-6	IL-1β	IL-17
SIRT3					
Pearson’s correlation	1	−0.051	−0.148	−0.303	−0.199
p		0.581	0.109	0.001	0.031
CRP					
Pearson’s correlation	−0.051	1	0.037	−0.088	0.031
p	0.581		0.687	0.341	0.741
IL-6					
Pearson’s correlation	−0.148	0.037	1	0.185	0.232
p	0.109	0.687		0.045	0.011
IL-1β					
Pearson’s correlation	−0.303	−0.088	0.185	1	0.262
p	0.001	0.341	0.045		0.004
IL-17					
Pearson’s correlation	−0.199	0.031	0.232	0.262	1
p	0.031	0.741	0.011	0.004	

### Predictive value of serum markers for POD in elderly patients undergoing general anesthesia

**Figure 2:**
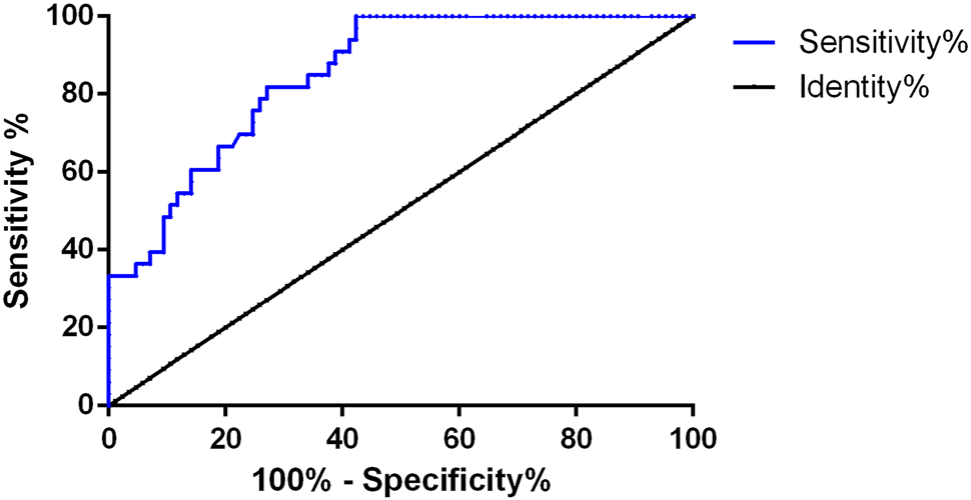
ROC curve of serum SIRT3 levels in POD patients.

We also assessed the ability of serum SIRT3 levels to predict postoperative delirium in elderly patients under general anesthesia using ROC curve analysis. As shown in Figure 2, the ROC curve indicated that serum SIRT3 could be a potential biomarker for predicting postoperative delirium. The AUC was 0.852, with a cutoff value of 20.01 ng/mL, sensitivity of 74.1 %, and specificity of 78.8 %.

### Logistic regression analysis of risk factors for POD in elderly patients undergoing general anesthesia

Initially, univariate logistic regression was used to analyze demographic data, clinical data, and serum markers as potential risk factors for POD. The results showed that anesthesia time, surgical time, ALB, Hb, SIRT3, IL-6, IL-1β and IL-17 were risk factors for POD in elderly patients undergoing general anesthesia (Table 3). Subsequently, significant variables identified in the univariate regression analysis were included in the multivariate binary logistic regression analysis, which revealed that ALB, SIRT3, IL-1β and IL-17 were risk factors for POD in elderly patients undergoing general anesthesia.

**Table 3:** Risk factors for POD in elderly patients undergoing general anesthesia.

Variables	Wald	Odds ratio	95 % CI	p-Value
Univariate				
Age	0.653	0.973	0.911–1.040	0.419
Sex	0.205	0.829	0.368–1.866	0.650
BMI	0.457	1.085	0.856–1.376	0.499
SBP	0.274	1.008	0.979–1.038	0.600
DBP	0.012	0.997	0.953–1.044	0.912
Intraoperative blood loss	0.172	1.012	0.957–1.069	0.678
ASA classification	0.582	0.830	0.515–1.338	0.446
Anesthesia time	8.287	0.969	0.949–0.990	0.004
Surgical time	5.123	0.973	0.951–0.996	0.024
Hb	5.248	1.045	1.006–1.086	0.022
ALB	23.448	1.494	1.270–1.757	<0.001
SIRT3	24.337	1.760	1.406–2.203	<0.001
CRP	0.298	1.002	0.995–1.008	0.585
IL-6	8.498	0.871	0.794–0.956	0.004
IL-1β	20.031	0.921	0.889–0.955	<0.001
IL-17	22.399	0.879	0.833–0.927	<0.001
Multivariable				
Anesthesia time	3.558	0.933	0.868–1.003	0.059
Surgical time	2.194	0.946	0.878–1.018	0.139
ALB	9.584	2.067	1.305–3.272	0.002
Hb	0.431	1.034	0.935–1.144	0.512
SIRT3	9.276	2.495	1.385–4.493	0.002
IL-6	2.379	0.777	0.563–1.071	0.123
IL-1β	4.157	0.920	0.849–0.997	0.041
IL-17	4.322	0.842	0.715–0.992	0.038

## Discussion

POD significantly impacted postoperative recovery, increased hospital stay, and costs [2], 15]. Therefore, it is urgent to develop early prevention strategies and provide comprehensive care for elderly patients at risk of developing POD. The findings of our research showed that serum SIRT3 levels could be used to predict POD in elderly patients undergoing general anesthesia surgery.

Increasing research has focused on the risk factors for POD, and our findings add new insights into this field. Iamaroon et al. reported that patients with pre-existing dementia and those aged 75 years or older were at the highest risk of developing POD, with an overall incidence of 11.6 % [16], 17]. Our results are consistent with these findings, as the majority of POD cases in our study occurred in elderly patients, indicating that aging-related vulnerability remains a major determinant of delirium. However, beyond age, our data further highlighted the importance of preoperative nutritional status. We found that patients who developed POD had significantly lower Hb and ALB levels before surgery, and ALB remained an independent predictor after multivariable adjustment. This aligns with the study by Chen et al. which demonstrated that a lower geriatric nutritional risk index (GNRI) was associated with a higher risk of POD [18]. Janssen et al. also identified renal impairment, smoking, and intensive care unit admission as risk factors in very old adults and observed that preoperative rehabilitation could reduce the occurrence of delirium [19]. In our study, these factors were uncommon, suggesting that nutritional and metabolic conditions may play a more dominant role in this relatively homogeneous elective surgical population. Similarly, a recent meta-analysis showed that age, diabetes, and intensive care unit stay were associated with increased POD risk [4], but in our cohort, these variables were not significant, implying that modifiable perioperative factors such as malnutrition and inflammation may have a greater influence on delirium development than chronological age. Furthermore, although anesthesia and surgery durations were longer in the POD group in univariate analysis, they were not independent predictors after adjustment, suggesting that their effects may be mediated through physiological stress and metabolic imbalance.

In our cohort, POD patients showed higher serum IL 6, IL 17, and IL 1β, together with lower serum SIRT3. Surgical stimulation can trigger inflammatory mediator release that perturbs neurotransmission and promotes delirium [20]. In elderly populations, increased IL 6, TNFα, and CRP have been linked to POD [21], and our results extend this pattern by implicating IL 17 and IL 1β. Mechanistically, SIRT family proteins can deacetylate and inhibit NF kappa B, lowering proinflammatory cytokine production and easing the inflammatory response [22], and SIRTs also restrain macrophage inflammatory signaling under LPS stimulation, with pharmacologic SIRT activators showing broad anti-inflammatory effects [23]. Consistent with these pathways, SIRT3 in our study was inversely correlated with IL 1β and IL 17, supporting a model in which reduced SIRT3 weakens anti-inflammatory control and permits an intensified cytokine milieu that favors POD.

In our study, serum SIRT3 levels were significantly lower in patients who developed POD, indicating that diminished SIRT3 levels was closely related to the onset of delirium. This finding suggested that reduced SIRT3 might have increased vulnerability to postoperative neuroinflammation and cognitive dysfunction. Mechanistically, SIRTs participated in delaying cellular aging and protected cells through inhibition of apoptosis, antioxidant stress, and maintenance of chromatin stability [24], 25]. They also showed neuroprotective effects in Parkinson disease and Alzheimer disease [26], 27]. In animal models of POD, increased SIRTs expression in the hippocampus and white matter improved cognitive function, reduced neuroinflammation, and decreased neuronal apoptosis [28], while SIRT3 prevented anesthesia and surgery related cognitive decline in aged mice by suppressing hippocampal neuroinflammation [29]. Taken together, our clinical data supported these experimental findings, and in multivariable logistic regression SIRT3 remained an independent protective factor for POD, with lower SIRT3 identifying patients at higher risk in this surgical population.

Furthermore, the ROC curve analysis showed that serum SIRT3 had good discriminatory ability for POD, with an AUC of 0.852, suggesting a favorable predictive performance. This result implied that SIRT3 might serve as a potential biomarker to help identify patients at increased risk of postoperative delirium. Considering its regulatory role in mitochondrial metabolism, oxidative stress, and inflammatory signaling, SIRT3 could possibly represent not only a predictive indicator but also a promising molecular target for future investigation. Enhancing SIRT3 activity might help improve neuronal energy metabolism and attenuate neuroinflammatory responses, thereby potentially reducing susceptibility to cognitive dysfunction after surgery. From a clinical perspective, perioperative assessment of SIRT3 levels might contribute to risk stratification and provide a reference for developing early preventive strategies in elderly surgical patients.

Of course, this study still has some limitations. Firstly, it is a single-center study with a small sample size. Secondly, only a limited number of cytokines were measured. Thirdly, further clarification is needed regarding the molecular mechanisms of SIRT3 in POD and the specific mechanisms by which SIRT3 inhibits inflammation. Fourth, we acknowledge that cytokines such as IL-6 may exhibit strong perioperative dynamics. Future studies including multiple sampling time points are warranted to better characterize their temporal profiles and relationship with POD development. In addition, the potential influence of the timing of surgery (daytime vs. nighttime operations) on delirium risk was not analyzed in our cohort, and this aspect should be explored in future research. Finally, our twice-daily assessments at standardized morning and evening time points were intended to cover potential diurnal fluctuations. Although the 72 h window aligns with prior perioperative studies in which delirium is commonly detected within the first few postoperative days, we acknowledge that extending assessments to postoperative day 4–5 could identify occasional late-onset cases, and this warrants future investigation.

## Conclusions

This prospective study found that serum SIRT3 levels were significantly lower and negatively correlated with the inflammatory markers IL-6 and IL-17 in elderly POD patients. Serum SIRT3 can predict the development of POD in these patients and acts as a risk factor for its occurrence.
